# Role of Platelet-Derived Microvesicles As Crosstalk Mediators in Atherothrombosis and Future Pharmacology Targets: A Link between Inflammation, Atherosclerosis, and Thrombosis

**DOI:** 10.3389/fphar.2016.00293

**Published:** 2016-08-31

**Authors:** Lina Badimon, Rosa Suades, Eduardo Fuentes, Iván Palomo, Teresa Padró

**Affiliations:** ^1^Cardiovascular Research Center, Consejo Superior de Investigaciones Científicas – Institut Català de Ciències Cardiovasculars, Institut d’Investigació Biomèdica Sant Pau, Hospital Santa Creu i Sant PauBarcelona, Spain; ^2^Cardiovascular Research Chair, Universitat Autònoma de BarcelonaBarcelona, Spain; ^3^Department of Clinical Biochemistry and Immunohematology, Faculty of Health Sciences, Interdisciplinary Excellence Research Program on Healthy Aging, Universidad de TalcaTalca, Chile; ^4^Centro de Estudios en Alimentos Procesados, Conicyt-RegionalGore-Maule, Talca, Chile

**Keywords:** atherosclerosis, cardiovascular diseases, cell-derived microvesicles, inflammation, platelets, thrombosis

## Abstract

Reports in the last decade have suggested that the role of platelets in atherosclerosis and its thrombotic complications may be mediated, in part, by local secretion of platelet-derived microvesicles (pMVs), small cell blebs released during the platelet activation process. MVs are the most abundant cell-derived microvesicle subtype in the circulation. High concentrations of circulating MVs have been reported in patients with atherosclerosis, acute vascular syndromes, and/or diabetes mellitus, suggesting a potential correlation between the quantity of microvesicles and the clinical severity of the atherosclerotic disease. pMVs are considered to be biomarkers of disease but new information indicates that pMVs are also involved in signaling functions. pMVs evoke or promote haemostatic and inflammatory responses, neovascularization, cell survival, and apoptosis, processes involved in the pathophysiology of cardiovascular disease. This review is focused on the complex cross-talk between platelet-derived microvesicles, inflammatory cells and vascular elements and their relevance in the development of the atherosclerotic disease and its clinical outcomes, providing an updated state-of-the art of pMV involvement in atherothrombosis and pMV potential use as therapeutic agent influencing cardiovascular biomedicine in the future.

## Introduction

Cardiovascular diseases (CVD) result in more than 19 million deaths annually and coronary heart disease (CHD) accounts for the majority of this death toll. In most cases, atherosclerosis and thrombosis associated with unstable plaques are the major cause for cardiovascular events (CVEs) including acute coronary syndromes (ACS) and stroke (van der Wal and Becker, 1999). Atherosclerosis is a systemic disease that starts early in life, asymptomatically progressing though adulthood, until clinically manifested. Indeed, large numbers of subjects who die suddenly of CHD are apparently healthy and had no previous symptoms (Mujica et al., 2010). Atherothrombosis is regulated by both genetic and environmental factors (e.g., dyslipidemia, hypertension, smoking, diabetes, and obesity; Marenberg et al., 1994; Palomo et al., 2006). The development of atherosclerotic lesions result from a complex interplay between circulating factors and various cell types in the vessel wall, which leads to the accumulation of lipids in the subendothelial space and a complex process of chronic inflammation, mainly characterized by endothelial dysfunction, leukocyte infiltration, and platelet activation (van der Wal et al., 1994; Nishijima et al., 2004). Indeed, platelet activation and subsequent platelet aggregation processes play an essential role in the development of atherosclerosis, possibly through a vast amount of molecules released upon platelet activation (Palomo et al., 2008). Interestingly, increasing evidence support the view that the role of platelets in atherosclerosis and its thrombotic complications may be mediated, in part, by local secretion of molecular effectors embedded or packed into microvesicles from the platelet surface.

Circulating microvesicles (cMVs) may participate in haemostatic and inflammatory responses, neovascularization, cell survival, and apoptosis, processes which are involved in atherothrombosis (Aatonen et al., 2012). Thus, increased levels of cMVs derived from platelets (pMVs), erythrocytes (ErMVs), leukocyte (LMVs), and endothelial cells (eMVs) are associated with individual metabolic abnormalities caused by metabolic syndrome and oxidative stress (Ueba et al., 2008; Helal et al., 2011). ErMVs, LMVs, and eMVs seem to be more abundant in human atherosclerotic plaques than in plasma (Leroyer et al., 2007). High amounts of these plaque MVs result from apoptotic leukocytes within vulnerable plaques. In contrast, MVs of platelet origin are the most abundant in blood (Rank et al., 2010). Circulating MVs are found in the plasma of healthy subjects (Horstman and Ahn, 1999; Berckmans et al., 2001; Caby et al., 2005; Toth et al., 2007; Grant et al., 2011; Herring et al., 2013) although their relative concentrations are determined by the pathophysiological context. Background levels of circulating pMVs in the absence of disease likely originate from aging platelets in the absence of activation (Cauwenberghs et al., 2006). The local release of MVs from platelets at the site of platelet plug formation indicates a possible role of pMVs in the haemostatic response *in vivo* (Lubsczyk et al., 2010). pMV membrane is a composite of the platelet plasma- and granule membranes (Biro et al., 2005) and have procoagulant properties which lead to thrombin generation. Such procoagulant activity (PCA) relies on the exposure of membrane anionic phospholipids that enable the assembly of coagulation complexes at the MV surface, and on the eventual thrombin formation (Sinauridze et al., 2007). Besides the well-known role of platelet-derived MVs in coagulation, thrombosis, and haemostasis, pMVs have been involved in a variety of processes such as wound healing, inflammation, CVD, diabetes, arthritis, tissue regeneration, and cancer.

This review summarizes and highlights the latest findings of the complex cross-talk between platelet-derived microvesicles, inflammatory cells, and vascular elements, and provides novel insight and understanding in the development of the atherosclerotic and thrombotic disease as well as potential clinical application of pMVs in diagnosis and therapy.

### Types of Microvesicles

All blood cells are able to release small membrane bound vesicles. Extracellular vesicles (EVs) are a heterogeneous population of membrane-coated microvesicles released by several cell types upon activation or apoptosis (Azevedo et al., 2007), and include plasma membrane-derived microparticles or microvesicles, multivesicular body-derived exosomes, and apoptotic bodies. These different types of EVs vary in size, and in phospholipid, nucleic acid, and protein composition (VanWijk et al., 2003).

*Microparticles* or *microvesicles*, which directly originate from the membrane surface, are characterized by phosphatidylserine (PS) exposure and are generally referred to be between 0.1 and 1.0 μm of diameter (Wiedmer et al., 1990). MVs, with densities between 1.04 and 1.07 g/mL, are of irregular shape and very heterogeneous in size. In contrast to MVs, *exosomes* (20–100 nm) are cup-shaped vesicles released from exocytosis of endocytic multivesicular bodies, with a density of 1.10–1.18 g/mL, and the distinction between both types of vesicles is complex due to an overlap in their molecular properties and sizes. Exosomes were first described in platelets, in which the differentiation with MVs is complex because of α-granules, and in general form a more homogenous population than MVs, both by size and molecular content. Thus, multivesicular bodies, the source of exosomes, are also considered to be pre-stages of α-granules (van Nispen tot Pannerden et al., 2010), which may then liberate exosomes on fusion with the plasma membrane. However, several α-granule-derived molecules are also present on pMVs. Moreover, the common exosomal marker tetraspanin CD63 is not only enriched in the platelet-derived exosomes, but it is also present on pMVs (van der Zee et al., 2006) and, in its turn, many common pMV proteins are detected on subsets of platelet exosomes (Heijnen et al., 1999). *Apoptotic bodies*, remnants of dead cells in the process of their shrinkage and elimination tend to be larger than MVs, may contain different internal components [e.g., deoxyribonucleic acid (DNA), organelles] and do not present prothrombotic activity despite displaying PS (Jimenez et al., 2003; Hristov et al., 2004). They present a density of 1.24–1.28 g/mL and their role is still not clear. Apoptotic bodies might be an easier system for cellular clearance themselves due to small size or, alternatively, an active signal to promote cellular clearance of the remaining damaged cells (Wickman et al., 2013).

### Platelet-Derived Microvesicle Release and Clearance

Platelet-derived microvesicles represent about 70–90% of all circulating MV pool and contribute to many biological processes (Berckmans et al., 2001; Aatonen et al., 2012). pMVs are elevated in peripheral blood as a result of chronic platelet activation in various disease states (Tan et al., 2005). Exposure of native blood to very high shear rate increases platelet expression of GPIIb/IIIa, rendering the platelet membrane procoagulant and also stimulating pMV formation (Sakariassen et al., 1998). Indeed, previous studies with patients with genetic defects have shown that impaired platelet PCA is translated into reduced vesicle formation (Sims et al., 1989; Gemmell et al., 1993). Despite the mechanisms by which MVs are formed are not well-known yet, pMVs are specifically shed from the platelet plasma membrane by an exocytic budding process (Holme et al., 1993), which involves increases in intracellular calcium, cytoskeleton reorganization (Yano et al., 1994; Pasquet et al., 1996) and changes in membrane lipid asymmetry, and is triggered by physical stimuli (shear stress, hypoxia) (Gemmell et al., 1993; Takano et al., 2004), by a variety of specific agonists (in an additive or even synergistic way; Xiao et al., 2002) or by platelet prolonged storage without agonist/stimuli requirement (Owens, 1994). While most non-physiologic agonists like calcium ionophore are the most potent inducers of MVs, the order of potency of physiologic agonists is C5b-9 membrane attack complex >thrombin plus collagen >thrombin >collagen >adenosine diphosphate >epinephrine (Connor et al., 2010). Other platelet stimuli are proinflammatory mediators [like lipopolysaccharide (Stahl et al., 2011), cytokines (Nomura et al., 2000), and soluble CD40 ligand (Prasad et al., 2003)], PAR agonists (Chung et al., 2004), thrombin receptor activating peptide (Tschuor et al., 2008), and prolyl gallate (Xiao et al., 2002), among others. Regarding ADP, the P2Y12 receptor contributes to pMV formation from activated platelet surfaces without any significant involvement of the P2Y1 receptor (Kahner et al., 2008).

Clearance of pMVs remains unknown. In contrast to platelets that have a life span of about 10 days, pMV half-life is about 30 min in mice (Flaumenhaft, 2006), or even less than 10 min in rabbits (Rand et al., 2006). In other circumstances, such as insertion of material test segments to a shunt in canines, pMV clearance is delayed to a 3–24 h period following removal of test segment from the shunt (Gemmell et al., 1997). Recently, aphaeresis-derived pMVs were shown to circulate for more than 5 h (Rank et al., 2010), increasing the discrepancy in pMV turnover, which may rely on distinct fate signals due to secretion process (Dasgupta et al., 2009; Abdel-Monem et al., 2010). Indeed, levels of pMVs might reflect the balance between dynamic mechanisms for release and clearance (Ayers et al., 2015).

## Platelet-Derived Microvesicles

Platelet-derived microvesicles retain certain parental cell characteristics, such as surface receptors, although their content depends on platelet stimuli, giving differences in their phenotype (Sims et al., 1989; Montoro-Garcia et al., 2014). pMV protein content is highly dependent on the type of physiological agonists involved in platelet stimulation (Milioli et al., 2015). MVs were shown to differ in content between human samples and between MV size classes (Dean et al., 2009; Bastos-Amador et al., 2012). Regarding phospholipid MV content, the relative abundance of lyso-phosphatidylcholines among other phospholipid classes (phosphatidylcholines, lyso-phosphatidylethanolamines, PSs, phosphatidylinositols, sphingomyelins, and diacyl- and plasme(a)nyl-phosphatidylethanolamines) was found to be significantly higher in blood MVs than platelets and platelet-derived MVs (Losito et al., 2015). In terms of protein composition, pMVs from activated platelets are highly enriched in α-granule-derived factor Va and Xa (Monkovic and Tracy, 1990) and thus possess procoagulant properties (Connor et al., 2009). Despite their heterogeneity (Perez-Pujol et al., 2007) a high percentage of pMVs express surface activation markers such as glycoprotein (GP)-Ibα (CD42b), P-selectin (CD62P), β_3_-integrin (GPIIIa, CD61), α_IIb_-integrin (GPIIb, CD41), lysosomal-associated membrane protein-3 (LAMP3, CD63), and thrombospondin-1 (TSP-1). However, healthy individuals may also have CD41^+^/CD61^+^/PS^+^-MVs derived from megakaryocytes instead of activated platelets. Megakaryocyte-derived MVs differ from pMVs in that they do not express granule fusion markers (CD62P^-^, LAMP-1^-^) and only contain full-length filamin A (Flaumenhaft et al., 2009). pMVs carry as well other platelet proteins such as platelet activating factor (PAF), β-amyloid precursor, anticoagulant protein C/S, complement C56b-9, and the chemokines CXCR4, CXCL4, CXCL7, and CCL5, which may exert potent biological effects in recipient cells of the circulation (Garcia et al., 2005). Indeed, pMVs were able to transfer fully operational surface receptors, such as CSCR4 and CD41, onto the recipient cells (Rozmyslowicz et al., 2003). pMVs thereby might modulate platelet, leukocyte, and vascular endothelial cells (EC) function, by modulating monocyte-EC interactions (Barry et al., 1998) and inducing chemotaxis (Barry et al., 1999). Because pMVs circulate in the blood flow, they could serve as shuttle modules and signaling transducers not only to neighboring cells (local environment) but also to cells at relative distance from their cell or site of origin, triggering cell activation, phenotypic modification, and reprogrammation of cell function, complementing the well-known processes of intercellular communication. How MVs are selectively released and targeted to exert their various pathophysiological functions remains unknown.

## Platelet-Derived Microvesicles and Cardiovascular Disease

Plasma levels of circulating MVs are known to increase with the presence of cardiovascular risk factors [hypercholesterolemia (Suades et al., 2014), obesity (Murakami et al., 2007), hypertension (Preston et al., 2003), diabetes mellitus (DM; Sabatier et al., 2002; Zhang et al., 2013), metabolic syndrome (Helal et al., 2011), sleep apnea (Trzepizur et al., 2014), and progression of atherosclerosis (Nomura et al., 1995; Ueba et al., 2008)]. Within DM, type-1 DM patients had twice tissue factor (TF)-positive pMVs than type-2 DM patients (Chiva-Blanch et al., 2016d). Likewise, elevated pMVs are associated with the 10-year CVD risk score (Ueba et al., 2010) and with age (van der Zee et al., 2006) in healthy men. Indeed, total number of pMVs was significantly greater in women than men in a population of healthy individuals (Gustafson et al., 2015). During past decades, the involvement and importance of the immune system and inflammation in the initiation and progression of atherothrombosis has become clear, also contributing to CVD risk. Raised levels of circulating pMVs have been detected in young women with polycystic ovary syndrome (Willis et al., 2014), in patients with rheumatoid arthritis in which pMVs express CLEC-2 (Gitz et al., 2014) and in patients with antiphospholipid antibodies in which pMVs correlate with anti-β2-GP (Chaturvedi et al., 2015). Additionally, elevated levels of circulating pMVs contribute to the increase PCA in patients with oral cancer (Ren et al., 2015).

High concentrations of circulating pMV (ranging from ∼3,000–11,000/μL) have been reported in patients with CHD including ACS (Michelsen et al., 2008; Bulut et al., 2009; Biasucci et al., 2012; Cui et al., 2013; George et al., 2015). In addition, circulating pMVs have been reported to reflect the size of myocardium at risk in patients with ST-elevation myocardial infarction (Jung et al., 2012) and correlated with the presence of atherothrombotic lesions in carotid plaques (Lukasik et al., 2013), intracranial atherosclerotic lesions (Kuriyama et al., 2010), early stage of coronary artery calcification in menopausal women (Jayachandran et al., 2008), cerebrovascular disease (Chiva-Blanch et al., 2016b) and peripheral arterial disease (van der Zee et al., 2006). Stenting in stable coronary atherosclerotic lesions is associated with a substantial release of pMVs (Horn et al., 2015). Interestingly, the link with increased circulating pMV levels is more consistent with coronary artery disease patients than in those with chronic kidney disease (Chen et al., 2014). Indeed, overproduction of pMVs and platelet activation with suppressed aggregation may be even implicated in the pathogenesis of coagulation abnormalities in children with congenital heart disease (Horigome et al., 2002; Ismail and Youssef, 2012). Recently, pMVs have been point out as biomarker of the vaso-occlusive phenotype-related severity in sickle cell anemia (Nebor et al., 2014). Furthermore, pMVs seem to be involved in the development of disseminated intravascular coagulopathy in critically ill patients but are not related to hospital mortality, while pMV/platelet ratio is independently associated to hospital mortality (Ohuchi et al., 2015). Finally, total pMVs were lower while activated platelet-derived MVs (P-Sel^+^) were higher in preeclampsia than in healthy women, being involved in the hypercoagulable intravascular reaction during pregnancies complicated by preeclampsia (Campello et al., 2015). Therefore, pMV may reflect the severity of the endothelial injury and platelet activation during thrombotic events (Tan et al., 2005; Jung et al., 2012).

Acquiring knowledge about the role of pMVs in CVD might also have implications for treatment. Caution must be taken when considering cMVs as pathological markers, since medical therapy with pharmacological agents can affect MV release during disease progression (Mobarrez et al., 2011; Nomura et al., 2011). Thus, pMVs are susceptible targets for pharmacological modulation and offer new options for therapies specifically focused on lowering MV levels. For instance, anti-platelets drugs such as GPIIb/IIIa inhibitors (Goto et al., 2003; Morel et al., 2004), acetilsalicilic acid (Bulut et al., 2011), and clopidogrel (Judge et al., 2010; Franca et al., 2012), and the anti-diabetic drug ticlopidine (Nomura et al., 2004c) have shown to reduce pMVs. In patients under antithrombotic treatment, pMVs exposing CD62P or CD142 are still elevated 6 months after initiation of the therapy (Skeppholm et al., 2012), possibly due to the fact that low-dose of acetilsalicilic acid might not be strong enough to suppress shedding of pMVs into the microcirculation (Lubsczyk et al., 2010). Our group has reported that aspirin intake in diabetic patients has no effect on pMVs (Chiva-Blanch et al., 2016d). However, statins as the cornerstone drug therapy for lipid-lowering decrease pMVs in patients with different cardiovascular risk factors. Thus, simvastatin and pravastatin decreased pMVs in patients with hypertension (Nomura et al., 2004a) and type-2 diabetes (Nomura et al., 2004b; Sommeijer et al., 2005). Similarly, atorvastatin reduces thrombin generation and expression of TF, GPIIIa, and CD62P on pMVs in patients with peripheral vascular disease (Mobarrez et al., 2011) and with type-1 diabetes and dyslipidemia (Tehrani et al., 2010). In a recent study aimed to evaluate the effects of lipid-lowering treatment on cMV generation in patients in primary prevention of atherosclerosis, we have demonstrated that, in hypercholesterolemic patients, statin treatment reduces not only the number of pMVs but markers of activated platelets, activated inflammatory cells and TF with respect to untreated subjects even when LDL levels were similar (Suades et al., 2013). In agreement, stroke patients with hyperlipidemia presented a significantly lower percentage of pMVs compared to control subjects, not only due to lipid-lowering but also because of a significant role in reduction of platelet activation and reactivity (Pawelczyk et al., 2015). Hence, in view of all these data statins may exert beneficial effects by inhibiting microvesicle generation and the triggering of MV-dependent mechanisms. Other cardioprotective agents that have an impact on pMV levels include calcium channel blockers (Nomura et al., 2005a,b), antioxidants such as vitamin C (Morel et al., 2003), and PPAR-pan agonists like bezafibrate (Kagawa et al., 2001). As several therapeutic drugs seem to influence the levels and composition of pMVs, the lowering of pMV load in the circulation might prove, at least in part, to be a novel therapeutic strategy for treatment. Nevertheless, whether the beneficial effect of a pharmacological approach is associated to pMV reduction and to a clinical improvement needs to be fully demonstrated.

In addition to pharmacological modulation, the therapeutic potential of progenitor cell-derived microvesicles is promising since they are naturally occurring, efficient, therapeutic delivery vehicle that might be used to deliver drugs to specific targets. Furthermore, the therapeutic potential of MVs has also been pointed out by the use of synthetic MVs, mimicking natural ones. MVs could have a broad potential in several conditions from inflammation to MI (Getts et al., 2014). Further characterization of the biological effects of these MVs is warranted.

## Platelet-Derived Microvesicles in Atheroinflammation and Atherothrombosis

Platelets are not only key mediators of thrombosis but also of inflammation by directly interacting with cells of the immune system (Fuentes et al., 2013). Reports in the last decade have described the secretion by platelets of proinflammatory molecules that exacerbate the inflammatory response in atherosclerotic lesions, during the initial injury to the endothelium as well as in the later stages when the atherosclerotic plaque is destabilized (von Hundelshausen and Weber, 2007). In addition to their pivotal role in CVD event presentation, platelet-derived MVs participate in inflammatory responses because they carry immune complexes, which are highly proinflammatory (Boilard et al., 2010; Cloutier et al., 2013). High-shear-stress-induced pMVs in a cone-plate viscometer device *in vitro* enhance expression of inflammatory cytokines either in ECs or in the human monocytic THP1-cell line (Nomura et al., 2001). Of note, pMVs may have a role in initial stages of atherosclerotic process, as they can facilitate cell-to-cell communication and adhesion processes between blood and vessel wall (Mause et al., 2005). pMVs could also play part in atherogenesis by enhancing proliferation of vascular smooth muscle cells (SMCs) as well as EC and chemotaxis and proliferation of hematopoietic cells (Miyazono et al., 1985; Weber et al., 2000; Baj-Krzyworzeka et al., 2002; Pakala, 2004). These results clearly suggest that pMVs contribute to atherosclerosis development and to vascular damage occurring in inflammatory disorders (Nomura et al., 2001; Inoue et al., 2006; Suades et al., 2015b). To this respect, Csongrádi et al. (2011) have described that blood pMV levels positively associate with abnormal carotid IMT and other risk factors in obesity suggesting a critical role of enhanced platelet reactivity in atherosclerotic wall alteration. In addition to pMVs, circulating lymphocyte-derived CD45^+^/CD3^+^-MVs have recently been shown to be biomarkers of asymptomatic subclinical lipid-rich atherosclerotic plaques in patients with familial hypercholesterolemia (Suades et al., 2014), and CD11b^+^-LMV of unstable plaques in asymptomatic patients with high-grade carotid stenosis (Sarlon-Bartoli et al., 2013). Indeed, CD45^+^/CD3^+^-lymphocyte-derived MVs were found increased in individuals at high cardiovascular risk (HCVR) who were about to develop a major CVE (Chiva-Blanch et al., 2016c).

In a similar fashion, levels of pMVs were significantly higher in patients with intermediate coronary lesions compared to subjects with normal coronary arteries (Chou et al., 2014). Progression of early atherosclerotic lesions to advanced plaques and their thrombotic complications are consequence of complex interactions between blood cells and arterial vessel wall components (Badimon and Vilahur, 2014). Erosion, fissure, or rupture of the atherosclerotic plaques are triggering events in ACS, being platelets a key player in these atherothrombotic processes (Fuster et al., 1990). We have found that HCVR patients have significantly high shedding of cMVs carrying epitopes of platelet activation and directly associate to lipid-rich subclinical atherosclerotic burden (Suades et al., 2015a). Besides, these prothrombotic pMVs have demonstrated an incremental prognostic value beyond the classical risk factor model for the prediction of cardiovascular risk, indicating that the state of activation of platelets in the blood-vascular interface may increase the release of pMVs that become markers of the high atherothrombotic risk (Suades et al., 2015a). Blood thrombogenicity can be partially explained by the fact that TF is not only present in the subendothelium, but also in the circulation (Sambola et al., 2003). TF is associated with monocytes, platelets, and even microvesicles, the latter represent an important source of the so-called blood-borne TF. We have also detected higher numbers of TF-positive MVs derived from platelets in HCVR patients; thus, pMV-associated TF might contribute to atherothrombosis. In the same line, our group has reported that clustering the information provided by TF^+^-pMVs, eMVs, and LMVs might predict CVEs in high-risk patients following a Mediterranean diet supplemented with nuts (Chiva-Blanch et al., 2016a).

Besides a potential relevance of MVs and pMVs as markers of subclinical atherosclerosis with a critical importance in reclassification of asymptomatic subjects, the follow-up study performed by Namba et al. (2007) in patients with a first ACS points to the fact that high pMV levels at discharge may also be an independent predictor for secondary thrombotic events and poorer clinical outcomes at 1 year. This study stresses the usefulness of pMV levels to differentiate patients who develop secondary atherothrombotic events from patients who develop a stable phenotype after a first ACS (Namba et al., 2007).

As pre- and analytical methodological procedures (Aatonen et al., 2014; Eckstein et al., 2014; out of the scope of this review) are still under assessment, care should be taken when expanding findings to prospective clinical studies. Prior to large-scaling, it would be desirable a complete international standardization of cMVs analysis.

**FIGURE 1:**
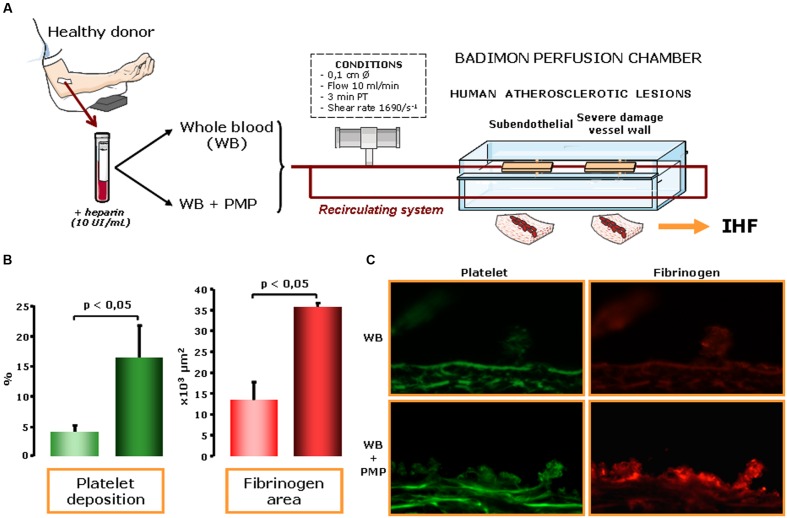
**Platelet-derived microvesicles (pMVs) in human blood enhance thrombosis on atherosclerotic plaques. (A)** Effects of pMVs on platelet deposition were assessed under controlled flow conditions exposing damaged arterial wall in the Badimon perfusion chamber to human blood immunohistofluorescence (IHF). **(B)** Platelet deposition on human atherosclerotic vessel wall was significantly increased in pMV-enriched bloods (6,000/μl). Specifically, pMVs also induced increase in platelet (*P* < 0.05) and fibrin (*P* < 0.05) deposition on human atherosclerotic arteries. **(C)** Immunofluorescence staining clearly showed the effects of adding pMVs to normal blood both in platelet and fibrinogen deposition. Representative immunophotographs of perfused human atherosclerotic substrates: platelet (green) and fibrin (red) deposition on human atherosclerotic vessels at a shear rate condition of 1,680 s^-1^ of whole blood and of whole blood with 6,000 pMVs/μl. Scale bar is 25 μm. Therefore, an increased content of pMVs, even in normal blood conditions, enhance platelet deposition, and thrombus formation.

Further to their potential as biomarkers of cell activation, increasing evidence support the concept that pMVs are causal inducers of atherosclerosis progression and thrombosis. MVs from human atherosclerotic plaques are highly abundant and more thrombogenic than plasma MVs, with differences between LMVs and pMVs (Leroyer et al., 2007). Ramacciotti et al. (2009) using a model of venous thrombosis, provided evidence that thrombus weight correlated negatively with LMVs and positively with pMVs. Besides, levels of pMVs have been found to be higher in culprit coronary arteries than in peripheral arteries of STEMI patients (Suades et al., 2016). In this microenvironment, pMVs released by activated platelets may provide a new prothrombotic interface for fibrin formation between the circulating blood and the growing thrombus (Ando et al., 2002), since they are able to interact with fibrin (Siljander et al., 1996). Thus, it has been proposed that pMVs might play a key role *in vivo* causing thrombotic events, even without the direct involvement of platelets (Oberle et al., 2007). The response to this hypothesis was provided by demonstrating that pMVs, beyond being biomarkers of cell activation, have functional effects on cardiovascular atherothrombotic disease because they enhance platelet and fibrin deposition on atherosclerotic arterial wall (Suades et al., 2012). This proof of principle study was performed perfusing atherosclerotic vessel wall with blood with/without exogenously added pMVs and showed that high pMVs concentration was able to induce more platelet and fibrin deposition (**Figure 1**) (Suades et al., 2012; Mause, 2013). We have also found a decrease of pMVs with surface markers of adhesion and activation in the post-thrombus blood after perfusing the exposed thrombogenic surfaces and in STEMI-patients (Suades et al., 2015c). Therefore, our data reinforce pMVs in blood promotes platelet adhesion due to a high tendency to adhere, as previously reported (Forlow et al., 2000) and support their clear implication in the atherothrombotic process. Altogether, this growing body of evidence support the view that pMV dissemination and exposure of their procoagulant membrane to the extracellular matrix (Merten et al., 1999) at the site of endothelial injury or onto the forming fibrin (Siljander et al., 1996) may serve as an adhesion surface on adhered platelets and within the thrombus to enable thrombin generation and to further support recruitment of platelets (Suades et al., 2012) and leukocytes (Mause et al., 2005) stimulating platelet aggregation (Berckmans et al., 2001; Nomura et al., 2001; Raturi et al., 2008).

## Molecular and Cellular Mechanisms Relating pMVs with Atherosclerosis Progression and Thrombus Formation

**FIGURE 2:**
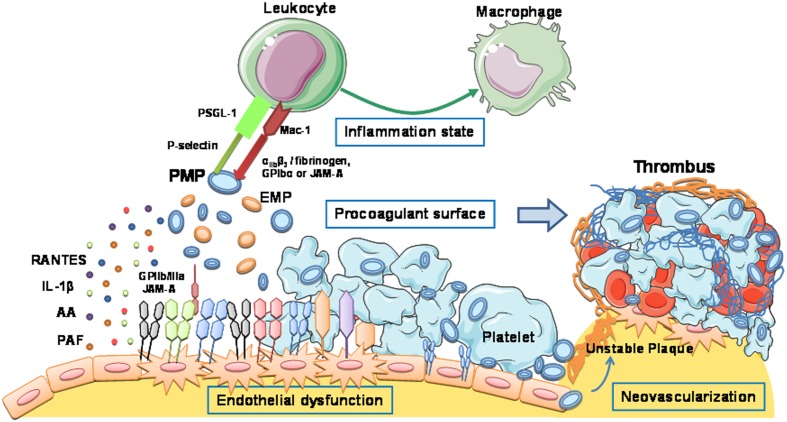
**Platelet-derived microvesicles promote vascular inflammation, atherosclerosis plaque progression, and thrombus formation**. Schematic representation of the molecular mechanisms by which MVs evoke atherothrombotic-related processes: endothelial dysfunction, proinflammatory reactions, procoagulant and prothrombotic effects, and neovascularization.

As described above, pMVs are considered to be both biomarkers and effectors of cell signaling. Importantly, circulating MVs can mediate communication between vascular cells because they allow membrane interactions between cells at distance (Meziani et al., 2008). Elucidation of the molecular mechanisms by which MVs might evoke and promote vascular inflammation, atherosclerosis plaque progression, and thrombus formation (**Figure 2**) is crucial for improving our understanding of their role in health and disease. Hereby, we present a compilation of functional studies investigating the underlying pMV-driven molecular processes in atherothrombosis by using either *in vitro, in vivo, or ex vivo models* (**Table 1**):

**Table 1:** Type of studies investigating the molecular mechanisms implicated in the effects of platelet-derived microvesicles in atherosclerosis progression and thrombus formation.

	*In vitro* models	*In vivo* models	*Ex vivo* models
Endothelial dysfunction	Boing et al., 2008 Merten et al., 1999 Sadallah et al., 2011 Xie et al., 2015	Merten et al., 1999	Boing et al., 2008

Neovascularization in atherosclerotic plaques	Battinelli et al., 2011 Brill et al., 2005 Italiano et al., 2008 Kim et al., 2004 Mause et al., 2010 Prokopi et al., 2009 Shai et al., 2012	Brill et al., 2005 Hayon et al., 2012 Ma et al., 2015 Mause et al., 2010 Ohtsuka et al., 2013 Shan et al., 2013 Varon et al., 2012	Ohtsuka et al., 2013

Proinflammatory activity	Baj-Krzyworzeka et al., 2002 Barry et al., 1997 Barry et al., 1998 Boudreau et al., 2014 Boilard et al., 2010 Brown and McIntyre, 2011 Brunetti et al., 2000 Cloutier et al., 2013 Dinkla et al., 2016 Forlow et al., 2000 Garcia et al., 2005 Inoue et al., 2008 Iwamoto et al., 1996 Jy et al., 1995 Kaneider et al., 2003 Lin et al., 2015 Lindemann et al., 2001 Mause et al., 2005 Mitsios et al., 2006 Miyazono et al., 1985 Nomura et al., 2001 Pakala, 2004 Ray et al., 2008 Sadallah et al., 2011 Setzer et al., 2006 Terrisse et al., 2010 Vasina et al., 2011 Vasina et al., 2013 Weber et al., 2000	Merten et al., 1999	Chiva-Blanch et al., 2016c Chou et al., 2014 Dinkla et al., 2016 Inoue et al., 2006 Inoue et al., 2008 Mause et al., 2005 Mitsios et al., 2006 Nomura et al., 2001 Sarlon-Bartoli et al., 2013 Suades et al., 2014 Suades et al., 2015b

Proacoagulant activity	Aleman et al., 2011 Berckmans et al., 2001 Butenas et al., 2005 Camera et al., 2010 Escolar et al., 2008 Forlow et al., 2000 Miyazaki et al., 1996 Gilbert et al., 1991 Lopez-Vilchez et al., 2007 Lösche et al., 2004 Raturi et al., 2008 Reininger et al., 2006 Sinauridze et al., 2007 Siljander et al., 1996 Suades et al., 2012 Suades et al., 2015c Tans et al., 1991	Chou et al., 2004 Oberle et al., 2007 Ramacciotti et al., 2009	Ando et al., 2002 Berckmans et al., 2001 Chiva-Blanch et al., 2016a Chung et al., 2007 Csongrádi et al., 2011 Hugel et al., 1999 Lopez-Vilchez et al., 2012 Namba et al., 2007 Raturi et al., 2008 Suades et al., 2015a Suades et al., 2015c Suades et al., 2016 Windelov et al., 2014

Neovascularization in atherosclerotic plaques	Battinelli et al., 2011 Brill et al., 2005	Brill et al., 2005 Hayon et al., 2012	Ohtsuka et al., 2013
	Italiano et al., 2008 Kim et al., 2004 Mause et al., 2010 Prokopi et al., 2009 Shai et al., 2012	Ma et al., 2015 Mause et al., 2010 Ohtsuka et al., 2013 Shan et al., 2013 Varon et al., 2012

### Platelet-Derived Microvesicles and Endothelial Dysfunction

Platelet-derived microvesicles pMVs interact with activated ECs and recruit activated platelets to injured subendothelium (Merten et al., 1999). T lymphocytes are recruited in the atherosclerotic lesion and an immunomodulatory role of pMVs on T cells has been recently described (Sadallah et al., 2014). pMVs carry a concentrated CD40L signal, induce polymorphonuclear leukocyte-damage of human pulmonary microvascular ECs and may affect the development of transfusion-related acute lung injury (Xie et al., 2015). Since MVs from platelets contain active executive caspase-3, they have also been involved in apoptosis (Boing et al., 2008).

### Platelet-Derived Microvesicles and Proinflammatory Activity

Platelet-derived microvesicle seem to be proinflammatory mainly through activating ECs (Merten et al., 1999) and enhancing cytokine responses (Barry et al., 1998). Thus, Barry et al. (1998), based on *in vitro* cell culture studies, described that pMVs activate ECs and monocytes, which results in increased adherence between both cell types. The effect of pMVs may relay in their content of unmetabolized AA and involve activation of PKC. Part of the proinflammatory and proatherogenic potential of pMVs has been related to their capacity to act as a transcellular delivery system for chemokines such as RANTES (CCL5) on activated vascular endothelium (outside-in signaling mechanism involving GP-IIb/IIIa and junctional adhesion molecule-A) triggering monocyte adhesion into early atherosclerotic lesions (Mause et al., 2005). Thus, MVs from stimulated platelets that are able to activate monocytes through RANTES, in turn facilitate monocyte migration, tissue recruitment and differentiation toward macrophage (Vasina et al., 2011).

In addition, pMVs promote expression of Von Willebrand factor (vWF) at the EC surface (involving anionic phospholipids, lactadherin, and GPIIb/IIIa) and the subsequent platelet/EC interaction under flow (Terrisse et al., 2010). pMVs also induce the expression of cyclooxygenase (COX)-2, but not COX-1 (Barry et al., 1997). Moreover, upon platelet activation and in response to lipopolysaccharide stimulation (TLR4/JNK/Akt pathway; Brown and McIntyre, 2011), a portion of IL-1β is shed in its mature form in pMVs and stimulates the adhesiveness of human ECs to leukocytes (Lindemann et al., 2001).

Other studies have shown that pMVs are also carriers of PAF (Iwamoto et al., 1996; Mitsios et al., 2006). Its presence in pMVs plays an important role in cell-to-cell interactions, as observed in models of acute and chronic inflammation (Mitsios et al., 2006). In this context, PAF production may be of great importance in coronary atherothrombosis and in the inflammatory response elicited during intracoronary injury induced by angioplasty (Goudevenos et al., 2001).

Interestingly, pMVs can also affect leukocyte aggregation and recruitment by direct interactions mediated by P-Selectin/PSGL-1-dependent interactions under flow conditions, especially in diseases where the concentration of the particles is elevated (Forlow et al., 2000), as well as Mac-1 (Inoue et al., 2008). In this regard, pMV binding to neutrophils can also increase neutrophil aggregation and phagocytic activity (Jy et al., 1995) and pMVs trigger monocytic cell aggregation and release of procoagulant TF-expressing MVs *in vitro* (Lin et al., 2015). In addition to pMV-mechanisms leading to inflammatory response, MVs released from thrombin-stimulated platelets to the extracellular space might contain mitochondria, which are able to interact with neutrophils triggering their adhesion to the endothelial wall (Boudreau et al., 2014). Additionally, pMVs from thrombin-activated platelets expressed CD40L and enhanced monocyte-derived dendritic cell maturation leading to the activation of T cells (Kaneider et al., 2003).

However, there are also conflicting reports in the scientific literature. pMVs inhibit IL-17 and IFN-γ production by regulatory T cells through P-selectin (Dinkla et al., 2016). pMVs also show inhibitory properties on macrophage and DC differentiation (Sadallah et al., 2011), emphasizing the concept of selective packaging of MV cargo dependent on platelet stimulus and the need to advance pMV characterization in future studies. Thus, pMVs actively take part in the immune response regulation at sites of vascular inflammation, where they are known to adhere and interact with leukocytes, promoting the healing process.

Furthermore, several lines of recent evidence support the concept that pMVs contain several miRNAs which may facilitate the communication between platelets with inflammatory cells (Gatsiou et al., 2012). For further details on this issue, please refer to review from Hulsmans and Holvoet (2013). All these crosstalk interactions are important for the propagation of inflammation at the site of vascular injury, as well as for sustaining thrombus growth (Santos-Gallego et al., 2014).

Finally, pMVs contain transcription factors, such as PPARγ, derived from parent cells (Ray et al., 2008). And proteomic analysis has led to the discovery of three other transcription factors in pMVs: RuvB-like 2, STAT3, and STAT5A (Garcia et al., 2005). pMV signaling induces differential expression of inflammation-relevant genes in monocytes, which represents a novel link between homeostasis and inflammation (Setzer et al., 2006). Besides, pMVs formed by aging platelets in an apoptotic-like process (Vasina et al., 2011) promote differentiation of monocytes to a resident CD14^+^/CD36^+^/CD68^+^-macrophage phenotype. These macrophage-like cells release metalloproteinases and H_2_O_2_ that contribute to plaque destabilization and eventual rupture, a clinically precipitating event in atherosclerotic disease (Vasina et al., 2013). Moreover, pMVs have shown to inhibit apoptosis of polymorphonuclear leukocytes (Brunetti et al., 2000). Therefore, specific bioactive pMV cargoes of miRNAs as well as of lipids and proteins might offer novel pharmacological targets for atherothrombosis therapy applicable in each stage of disease.

### Platelet-Derived Microvesicles and Procoagulant Activity

Although pMVs can express both procoagulant (Miyazaki et al., 1996) and anticoagulant proteins (Tans et al., 1991), a key feature of pMV is their procoagulant potential. High shear-stress rates and immobilized vWF on the luminal surface of an obstructing atherosclerotic plaque can trigger the generation of procoagulant MV via platelet GPIbα-vWF interactions (Reininger et al., 2006). Through membrane transverse migration and surface exposure of anionic phospholipids including PS, platelets release PS^+^-pMV that possess high affinity binding sites for activated coagulation factors such as factor IXa, Va, Xa, and VIII and provides a catalytic environment optimal for subsequent thrombin formation (Gilbert et al., 1991; Chou et al., 2004). Sinauridze et al. (2007), by comparing procoagulant properties of A23187-calcium ionophore-activated platelets and pMVs using several *in vitro* models of haemostasis, have provided evidence that pMV surface is approximately 50- to 100-fold more procoagulant than activated platelet surface. Low levels of procoagulant PS-positive pMVs are associated with impaired clot formation in trauma patients and may play an important role in trauma-associated coagulopathy (Windelov et al., 2014).

Another molecular property that conveys PCA to pMV is functional TF. As stated, the cellular origin of blood-borne TF is unresolved. LMV seem to display the most relevant amount of TF, the most important initiator of intravascular thrombin and fibrin formation (Lösche et al., 2004). Although TF cell origin is still controversial (Butenas et al., 2005), today is generally accepted that platelets carry and transfer TF (Camera et al., 2010) and also possess mechanisms to internalize TF-rich MVs (Escolar et al., 2008), and that platelet-associated TF enhances platelet reactivity and thrombin generation with flowing blood (Lopez-Vilchez et al., 2012). Increased TF-positive procoagulant MVs are present in the circulating blood of patients under pathological conditions (Hugel et al., 1999) and are taken up by platelets inducing aggregation in the presence of factor VII (Lopez-Vilchez et al., 2007). According to Lösche et al. (2004), pMV transfer TF to monocytes but not to neutrophils. In fact, *in vivo* appearance of TF-bearing pMV on leukocytes in pericardial blood during cardiac surgery has been reported (Chung et al., 2007). Other studies, however, describe a lack of TF in pMV because it is not packed in the platelet-derived MV during platelet activation (Aleman et al., 2011). This latter finding arise the concept that MV derived from monocytes and platelets exhibit unique PCA and differentially modulate clot formation, structure and stability. According to this hypothesis, monocyte-derived MV would initiate the extrinsic pathway whereas pMV would augment thrombin generation and promote clot propagation following TF- or contact-initiated clotting (Aleman et al., 2011). Further studies on *in vivo* thrombosis models are necessary to investigate the contribution of MVs from different parent cells to the thrombotic process.

### Platelet-Derived Microvesicles and Neovascularization in Atherosclerotic Plaques

A key factor in the evolution of subclinical atherosclerosis to an ischemic event is the increased vulnerability of atherosclerotic plaques. It is not known why some of the existing plaques in the arterial tree rupture and trigger thrombotic complications while some others do not. Human coronary plaques associated to ACS show the highest accumulation of neovessels (Juan-Babot et al., 2003). Increasing evidence shows that high density of neovessels in coronary atherosclerotic lesions is associated with hemorrhagic leaky vessels, unstable plaques, and high rate of thrombotic episodes (McCarthy et al., 1999). During plaque development pro-angiogenic pathways seem to be re-activated leading to formation of immature blood vessels prone to rupture. Infiltration of microvessels into the media, intima, and plaques, originates predominantly from proliferating *vasa vasorum* although recent work has also signaled toward bone marrow-derived circulating endothelial progenitor cells (EPC; Kawamoto et al., 2003). pMVs have shown to promote angiogenesis in a number of studies (Kim et al., 2004; Brill et al., 2005; Mause et al., 2010). *In vitro* cell culture studies provide evidence that pMV promote cell proliferation and survival, migration, and tube formation in human umbilical vein EC via GPCR and kinase signaling pathways (Kim et al., 2004). Similarly, pMV augment the adhesion and neovascularization capacities of circulating angiogenic cells obtained from atherosclerotic patients through a RANTES-mediated mechanism (Ohtsuka et al., 2013). pMVs induce sprouting both *in vivo* and *in vitro* (Brill et al., 2005) and influence the angiogenic activity of EPC (Prokopi et al., 2009). pMV-induced invasion of ECs through a layer of matrigel was mediated by vascular endothelial growth factor, heparanase, and platelet-derived growth factor, but not by basic fibroblast growth factor (Brill et al., 2005). Both Dok-2 and CD49f proteins participate in the mechanisms that regulate angiogenesis and, interestingly, are shown to be again differentially regulated in pMV depending on the platelet stimulus (Shai et al., 2012). These data are in line with the studies that show a differential localization of angiogenic cytokines in different platelet granules (Italiano et al., 2008) and that the release of angiogenic regulatory proteins is modulated by physiological processes (Battinelli et al., 2011), indicating by which mechanism operate the pro-angiogenic effect of pMVs. In a stroke model, a local delivery of pMV to the lateral ventricles induces angiogenesis, neurogenesis, and neuroprotection and reduces behavioral deficits after brain ischemia (Hayon et al., 2012; Varon et al., 2012).

Thus, in pathological states such as subclinical advanced atherosclerosis, pMV shed from the circulating platelets may reach adequate concentrations and that elevated levels of pMV could contribute to plaque development and instability. In a distinct scenario, pMVs could also collaborate with the remote conditioning protective effect against ischemic-reperfusion injury in a model of cerebral infarction, likely by exerting similar angiogenic properties (Shan et al., 2013). Indeed, ischemia-reperfusion preconditioning induces an increase in pMVs, which confer at least part of the remote protective effect against cardiac ischemic-reperfusion injury (Ma et al., 2015).

## Exosomes

Within EVs platelet-derived exosomes, first described by Heijnen et al. (1999), have received increasing attention due to their potential role as mediators of cell-to-cell communication. The presence of exosomes containing miRNAs in circulating blood, mainly derived from platelets, has emerged as a potential source of biomarkers of CVD. Additionally, they have been implicated in vascular and heart functions. Ago-miR-223 is delivered to EC via microvesicles from activated platelets (Laffont et al., 2013) and platelet-derived exosomes reduce ICAM-1 expression decreasing monocyte adhesion (Gidlof et al., 2013) and induce EC apoptosis (Janiszewski et al., 2004; Gambim et al., 2007). Thrombin-stimulated platelet-derived exosomes, enriched in miR-223, miR-339, and miR-21, inhibit PDGFR β expression in SMCs (Tan et al., 2016). Moreover, exosomes may serve as effectors by which damaged heart communicate for cardiac injury repair in the setting of MI (Ottaviani et al., 2016). It is not clear so far whether the intervention on exosome secretion processes during disease would have therapeutic effects. However, in the next upcoming years, study of exosomes molecular insights may help not only to detect novel biomarkers of disease but also to provide potential new therapeutic approaches for atherothrombotic cardiovascular disease.

## Conclusion and Perspectives

Platelet-derived microvesicles may not only act as a biomarkers of cell activation but also as important functional effectors that linking inflammation, hypercoagulability and neovascularization may contribute to the exacerbation of atherosclerotic lesion growth and to ongoing thrombosis. Thus, pMVs are emerging as novel and specific pharmacological targets to manage atherothrombosis and, additionally as potential therapeutic tools for drug delivery, cardioprotection, and regenerative and personalized medicine. Although our understanding of pMVs has considerably been expanded in the last decade, we are far from completely understanding platelet microvesicle biology. In the upcoming years, platelets and platelet-derived MV research will likely advance and take a leading position in tackling down CVD.

Further studies are required to improve our knowledge on the mechanisms underlying generation of circulating pMV, from the characterization of the specific phenotype of released pMVs by distinct stimuli and pathophysiological context, to the identification of the complete composition and its influence on other cells, and to the disentangling of molecular and signaling processes involving pMVs as functional effectors in atherosclerosis and atherothrombosis.

## Author Contributions

LB, IP, and TP conceived and coordinated the design of the review. LB, RS, and EF wrote the paper. RS and EF made figures; LB and RS edited the paper. All authors wrote part of the manuscript, provided critical comments, revised the manuscript and approved the final version of the manuscript.

## Conflict of Interest Statement

The authors declare that the research was conducted in the absence of any commercial or financial relationships that could be construed as a potential conflict of interest. The reviewer JZ and handling Editor declared their shared affiliation, and the handling Editor states that the process nevertheless met the standards of a fair and objective review.
